# Two Cases of Fracture; One of the Upper, the Other of the Lower Jaw

**Published:** 1792

**Authors:** T. Hughes

**Affiliations:** Surgeon at Stroud-Water in Gloucestershire.


VI. *Two Caſes of Fracture; one of the upper, the other of the lower Jaw. By Mr.* T. Hughes, *Surgeon at Stroud-Water in Glouceſterſhire.*

### CASE I.

ON the 22d of April, 1768, a lad, about ſixteen years of age, received ſo violent a kick in the face from a horſe as to throw him backwards.

backwards. The accident happened a mile or two from his maſter's houſe: he got up and walked home, bleeding all the way. I ſaw him a few hours after the accident, and found the upper jaw fractured. The fracture extended horizontally from the poſterior part of the right os maxillare, beyond the fartheſt tooth, as far as the cuſpidatus* on the left ſide, juſt above the alveolar proceſs, and nearly where the cheek and lip are connected to the gums. The inciſor teeth were beaten out, with the anterior part of their ſockets; and the poſterior part of the ſockets was detached from the other part of the fractured piece. The molares, bicuſpides, and cuſpidatus, of the right ſide, remained firm in their ſockets. The fracture extended alſo in an irregular manner through the bony palate, and the membranes were lacerated, ſo that the air ruſhed through the opening from the noſe into the mouth. By this extent of fracture all the alveolar proceſs of the right os maxillare, with its molares, bicuſpides, and cuſpidatus teeth, that part of the pterygoid proceſs of the os ſphenoides connected with it, and as much

* According to Mr. Hunter's arrangement in his Natural Hiſtory of the Human Teeth.

 of of the left os maxillare as form the fockets of its two incifors, with part of the bony palate, were beaten into the mouth, and made a very frightful appearance. He was very fenfible, and fpoke, but could hardly be underftood. The fractured piece was eafily reduced, but fell down immediately upon the fingers being removed, being held by the foft parts only.

I felt myfelf at a great lofs how to fupport the fracture, from the want of fome fixed point at the back part of the mouth, from not being provided with implements, and from its being late in the evening, in a country place, five miles diftant from Wotton-Underedge, my then place of refidence. I thruft fome compreffes between the teeth of the upper and lower jaw of the affected fide, and paffing a ligature through a convenient part of the bone near to the left cufpidatus, tied it to that tooth. By thefe means the piece was confiderably, though not fufficiently, raifed, efpecially the palatine part. The patient was put to bed, ordered to be kept cool and quiet, and to be fed with a liquid, laxative diet through the fpout of a tea-pot.

I found the next day, that, the compreffes and ligature having flipped, the fractured piece was fallen down; but an eryfipelatous tumour of of the face, attended with fever, having come on, nothing could be done in that ſtate to redreſs the fracture. Some blood was taken away, and a laxative mixture directed.

Two days afterwards Mr. Cooper, my partner, ſaw him with me. The eryſipelas being nearly ſubſided, we deliberated on the ſteps to be taken. While my thoughts were employed how to get over the difficulty of making a perforation through the firm part of the os maxillare, by which a ligature might be paſſed, and the fractured piece ſecured, Mr. Cooper, who ſeemed to be meditating ſome kind of prop, having raiſed the fractured piece with his finger, thruſt one end of the ſponge, with which we had been cleaning the parts, into the patient's mouth. We both immediately ſaw with great pleaſure that this ſubſtance, by its expanſibility, would effectually ſupport the parts in their proper ſituation. A piece, therefore, of a proper ſhape and ſize, ſo as moderately to fill the mouth, leaving ſufficient room on the left ſide for the ſpout of a tea-pot to be introduced for the conveyance of nouriſhment, and with a ſtring faſtened to it, was introduced. By this ſimple, eaſy method the fractured piece was ſo well ſupported in its proper ſituation, that,

 upon upon changing the ſponge thirty-ſix hours afterwards, the roof of the mouth was raiſed to its natural arch, which before could not be ſo well effected even with the finger.

The diſcharge of matter and mucus was ſo large and offenſive as to make it neceſſary afterwards to change the ſponge daily; and for this purpoſe, though the patient was much reduced, he received ſufficient ſupport from liquid nouriſhment to enable him to walk to Wotton from the village where he lived. After the firſt application of the ſponge, it was uſed in a dry ſtate, that it might abſorb more of the diſcharge. In about a fortnight the diſcharge leſſened, and the parts acquired a firmneſs ſufficient to enable him to change the ſponge himſelf, and in about a month there appeared to be no farther occaſion for it. The remaining poſterior part of the ſockets of the fore teeth were removed as they became looſe; as alſo a ſmall exfoliation from the palatine proceſs of the maxillary bone. The ſoft parts healed, and left no fiſſure in the palate.

By the uſe of the ſponge the fractured ſide of the upper jaw was ſo much raiſed, that the molares and bicuſpides teeth projected a trifle beyond their natural ſituation; but ſome time after after it had been laid aſide I obſerved that the teeth had fallen a little, ſo as to ſtand rather within the outer edge of the correſponding teeth of the lower jaw when the mouth was ſhut. From the ſame cauſe there was a ſlight depreſſion of that ſide of the face below the cheek bone. I obſerved alſo, that, from the violence committed by the accident, the left naſal bone, the lower edge of the orbit of the left eye, and the naſal proceſs of the left maxillary bone, were ſlightly raiſed: but from all this he could not be ſaid to have any deformity or defect, except what aroſe from the loſs of the fore teeth.

REMARK.

I do not know that any improvement could have been made in the uſe of the ſponge in this caſe, except that, inſtead of uſing it dry, it had been always ſqueezed out of water, or any other agreeable liquid, and changed twice or oftener in twenty-four hours, which this patient's ſituation did not admit of.

### CASE II.

In the evening of May 16, 1780, a boy, ſix years old, had his lower jaw fractured by a horſe, horfe, which was rode over him, and he was brought to me the next day. The fracture was nearly perpendicularly acrofs the jaw, betwixt the fecond incifor and cufpidatus teeth on the right fide, and from the pofterior part of the cufpidatus extended obliquely, behind the incifores and left cufpidatus, through the alveolar procefs and bafe of the jaw to the anterior part of the firft left molaris, in fuch a manner, that the anterior part of that portion of the jaw forming the chin and inclofing the four incifores and left cufpidatus teeth was fevered from the pofterior part, fo as to leave a large chafm between them, the gums being alfo torn, and forced downwards and to the left fide, fo as to be below its natural level from half or three quarters of an inch on the right fide to three quarters of an inch, or an inch, on the left fide, and to be about a finger's breadth diftant from the correfponding right fide of the jaw, and of courfe overlapping the left fide as much; the teeth in the detached piece remaining fixed in their fockets. The parts bled freely at the time of the accident, and the chafm between the fractured pieces was now filled with grumous blood.

It was not very difficult to bring the fractured pieces to correfpond on the right fide; but I could could not raiſe the depreſſed piece ſufficiently on the left to correſpond with that ſide of the jaw, owing partly, as it ſeemed, to the points of the fracture entangling each other, and partly to the action of the muſcles upon the boy's crying and endeavouring to ſhut his mouth in making the neceſſary attempts. No uſe could be made of ligatures to the teeth; and the uſual bandage was of no ſervice in ſupporting the depreſſed piece; however I left it on to keep the parts a little ſteady, and ordered that nouriſhment ſhould not be given by the mouth, but by clyſters, till I contrived and got made an apparatus more adequate to the purpoſe. This conſiſted of a couple of little irons, which acted on the principle of what mechanics call holdfaſts, to reſt on the teeth in the ſides of the jaw above, and fixed below and before, by means of ſcrews, to a caſe of copper, adapted to the form of the jaw, and ſecured by tapes faſtened to a cap on the head. This, properly defended, was applied on the 20th; at which time, beſides a conſtant flow of ſaliva, mucus, &c., from the mouth, there was conſiderable ſwelling and hardneſs under the jaw; and the leaſt attempt to hold back the head put him to great pain. Directions were given to keep the parts clean by frequently frequently injecting barley water with honey of roses and tincture of myrrh.

By means of the machinery, the depressed piece of jaw was at first kept in a much better situation, and the parts so steady, that the boy could move his head much more easily than before, and take liquids; and I hoped to derive great advantage from it. But although alterations were occasionally made in its form and application, I afterwards found that, from the difficulty of keeping the left holdfast far enough back, and of preventing one or other from slipping now and then, and from the lip swelling from the necessary pressure of the irons, circumstances which appeared to irritate and render him restless, and as a greater force applied with a view to support the depressed piece only increased the separation, by exciting the action of the muscles of the jaw, and as I saw that exfoliations would take place in the alveolar process; on all these accounts I laid aside the holdfasts, after using them four days, and continued the use of the copper case only.

I frequently thought of the advantage received from the sponge, in the case of the fracture of the upper jaw above related; but did not at first conceive that, in the present case, any advantage vantage could be reaped from it. However, after ſome days, it occurred to me, that a piece of dry ſponge, made flat by preſſure, and placed on the inſide of the copper caſe, by ſwelling from the conſtant flow of ſaliva, &c. from the mouth, might gradually raiſe the depreſſed piece of jaw, without occaſioning pain. This was applied on the 30th, a fortnight from the time of the accident. Having found nothing applicable to the caſe in authors, I related it to ſome gentlemen of my acquaintance of experience and abilities, who readily informed me of what they had done in ſomewhat ſimilar caſes, but which could not be uſed in the then ſtate of my patient, except wedges of cork placed between the teeth on the ſides of the jaws; I, therefore, tried them, with the ſponge under the chin at the ſame time. The wedges were uſed three days, cut in various forms, as ſeemed beſt to keep the fractured parts ſteady, and themſelves from ſlipping; but, I was obliged to give them up, as they occaſioned great pain, and either brought on or increaſed an hectic fever, attended with diarrhœa, by which the patient was ſo much reduced that his life was not expected. The uneaſineſs he ſuffered from the wedges ſeemed to ariſe, partly from their ſlipping ſometimes and thereby ſeparating the fractured pieces, but chiefly from their keeping the jaw in a depreſſed and unnatural ſtate. The left cuſpidatus tooth, by bearing againſt and overlapping the adjoining molaris, ſeemed to be another obſtacle to the raiſing of the depreſſed piece: I, therefore, removed the cuſpidatus, and then truſted to the power of the ſponge, applying a dry piece daily betwixt the chin and copper caſe, and occaſionally alſo keeping another bit of ſponge betwixt the right cheek and ſide of the jaw, (which was carried too much outwards), and continuing to waſh the parts with the injection. When this method was firſt uſed, the middle detached piece of the jaw was ſo much below its natural level, as eaſily to admit nouriſhment to be conveyed between the upper and lower teeth, by means of a tea pot with a flat ſpout, or a ſpoon.

In a few days he became quite eaſy, and his general health mended; alſo, the depreſſed piece of jaw was gradually raiſed, ſo that by the end of June the fore teeth of the lower approached thoſe of the upper ſo nearly, that he retained moſt of the ſaliva; and as it advanced, the fracture acquired firmneſs likewiſe to admit of his opening his mouth with eaſe, and receiving ſome ſolid food, brought to a ſtate not requiring maſtication.

maſtication. By the middle of July, there was neither waſte of ſaliva, nor any further occaſion for the ſponge. The copper caſe was uſed conſtantly a month longer; and afterwards, when he was eating, till the beginning of September. He began to maſticate ſolid food in the middle of Auguſt. From the middle of June to the middle of Auguſt, ſeveral pieces of the alveolar proceſs exfoliated; alſo a ſmall bit of the jaw was caſt off on the left ſide, through a ſmall opening it had produced in the teguments; and a tooth fell out, which proved to be the body of a cuſpidatus of the ſecond ſet, its fang not yet formed. During the cure, no advantage was received from internal medicine, as he could not be prevailed with to take any.

In September, the appearances of the parts concerned were theſe: When the mouth was ſhut, no mark of diſeaſe was diſcovered, except a ſlight pit in the teguments under the left ſide of the jaw, where the external exfoliation happened. On turning down the under lip, the teeth and gums were perfectly regular in the circular range; though the left cuſpidatus was deficient, there was no chaſm betwixt the inciſor and molaris; and there was ſo little diſtance betwixt the upper and lower inciſors, that it would would not have been obſerved unleſs pointed out. Indeed, the greateſt deformity was what aroſe from the fore teeth of the lower jaw being incruſted with tartar, from want of uſe.

From October to December he ſhed the milk inciſors; and, the firſt molaris on the left ſide having been looſened by an exfoliation, I removed it, and at the ſame time the crown of a bicuſpis of the ſecond ſet.

In June 1787, he then being thirteen years old, I had an opportunity of examining him. The right cuſpidatus tooth, and one of the bicuſpides on the left ſide were deficient, as could not otherwiſe be expected; the reſt of the teeth were very regular, more ſo than the teeth of perſons in general who have the full number. There was a ſmall chaſm, but not very ſtriking, betwixt the ſecond inciſor and the firſt bicuſpis on the right ſide. Though all his teeth were large, the bicuſpides on the right ſide, and the bicuſpis and cuſpidatus on the left, ſeemed to be larger than uſual. The teeth of the lower not only met thoſe of the upper jaw, but by carrying the jaw backwards, he could bring the lower behind the upper ones.

### REMARKS.

It is ſeen from the above account, that, in the reduction of the fracture, what could not be done at once by force, was brought about gradually and with eaſe; which mode of conduct may be advantageouſly purſued in other fractures, when difficulties oppoſe a ſpeedy reduction.

It appears alſo, that there was an imperfection in the cure, by the line of the jaw being ſhortened, and conſequently not leaving ſufficient room for the full number of teeth; but perhaps the cure was as perfect as the circumſtances of the caſe would admit. And, indeed, unleſs thoſe of the ſecond ſet of teeth, which were loſt, could have been ſaved; it was an advantage, in the event, to have the circle of jaw leſſened likewiſe, otherwiſe, there muſt have been chaſms between the teeth, which nature could not have filled up. On the other hand, had all the teeth been preſerved, unleſs the circle of the jaw could have been extended by keeping its ſides at a greater diſtance, the teeth muſt have ſtood in a very irregular manner.

*Stroud Water*,

May 8, 1792.

